# Towards Feasible Home ECG Monitoring: AI-Driven Detection of Clinically Critical Arrhythmias Using Single-Lead Signals

**DOI:** 10.3390/bioengineering13030317

**Published:** 2026-03-10

**Authors:** Chia-Hsien Hsu, Jui-Chien Hsieh, Po-Yuan Su, Chung-Chi Yang

**Affiliations:** 1 Laboratory of Medical Informatics and Telemedicine, Department of Information Management, Yuan Ze University, Taoyuan 32003, Taiwan; s1089201@mail.yzu.edu.tw (C.-H.H.); s1071644@mail.yzu.edu.tw (P.-Y.S.); 2 Division of Cardiovascular Medicine, Taoyuan Armed Forces General Hospital, Taoyuan 32551, Taiwan; t220979@gmail.com

**Keywords:** sinus bradycardia, sinus tachycardia, supraventricular tachycardia, ventricular tachycardia, deep learning

## Abstract

Differentiating life-threatening arrhythmias, such as ventricular tachycardia and supraventricular tachycardia, from non-threatening ones is crucial for clinical applications. This study aimed to develop a deep learning model to classify five key Electrocardiogram (ECG) patterns: normal sinus rhythm, sinus tachycardia, sinus bradycardia, supraventricular tachycardia, and ventricular tachycardia. We collected 1500 single-lead 10 s ECG signals from public datasets, including PhysioNet/Computing in Cardiology (CiC) Challenge 2020 and the Malignant Ventricular Ectopy Database, for training and 2297 ECGs for testing. Each 10 s signal was decomposed into 1 s sliding windows with a 5-point stride, which served as the input for the proposed deep learning architecture utilizing temporal attention and Time2Vec embedding. The model performance achieved an overall accuracy of 95.2%. For the five classes—supraventricular tachycardia, sinus tachycardia, normal sinus rhythm, ventricular tachycardia, and sinus bradycardia—the model achieved sensitivities of 90.3%, 92.9%, 97.4%, 100.0%, and 99.0% and accuracies of 96.3%, 95.8%, 98.9%, 99.9%, and 99.5%, respectively. Specificities for all rhythm categories exceeded 97.4%. This simple and effective single-lead model can significantly support the growing trend of home healthcare and professional clinical decision-making.

## 1. Introduction

At-home ECG monitoring solutions capable of accurately screening for both fatal and non-fatal arrhythmias are crucial for effective treatment and reducing medical errors [1,2]. This is particularly vital for non-cardiologists and home-care settings when managing life-threatening conditions like supraventricular tachycardia (SVT) and ventricular tachycardia (VT) [3,4,5]. Current research in automated ECG classification has primarily focused on single QRS waveform analysis using datasets such as the MIT-BIH Arrhythmia Database. However, these approaches often face limitations regarding subject diversity and the ability to discern overall rhythm patterns. While many deep learning (DL) models, including Convolutional Neural Networks (CNNs) and Recurrent Neural Networks (RNNs), have shown promise, capturing the complex, long-range dependencies inherent in time-series ECG signals remains a challenge [6]. Recently, Transformer models have emerged as a powerful alternative due to their self-attention mechanisms, which are well-suited for processing sequential medical data and capturing these long-range dependencies effectively [6,7,8].

### 1.1. Deep-Learning-Based ECG Classification

A common approach in the literature involves utilizing the MIT-BIH Arrhythmia Database, which predominantly features long-term recordings from a limited number of subjects [9,10]. However, a significant challenge in this field is the lack of clear data splitting strategies in many studies, as including the same patient in both training and testing sets can lead to overestimated performance [11,12,13,14]. Beyond data splitting, researchers have explored various processing techniques to obtain samples, including QRS detection, waveform segmentation, and fixed-time intervals [9,10,11,12,13,14,15,16,17,18,19,20,21,22,23], often implemented with DL models such as CNNs and RNNs [16,17,19].

Despite their successes, capturing long-range patterns in time-series ECG signals remains a hurdle [16,17,19]. Recent studies specifically targeting fatal arrhythmias have attempted to overcome this by utilizing segment-based analysis or multi-step frameworks to better distinguish between complex rhythms like SVT and sinus tachycardia (ST). Nonetheless, achieving high clinical generalization while maintaining sensitivity for rare, life-threatening events remains an ongoing effort in the field [16,17,19]. Consequently, Transformer models have recently emerged as a robust solution for large-scale classification because they can handle long sequences and capture complex temporal relationships [11,12,13,14,24,25,26].

As summarized in Table 1, a review of recent ECG research indicates an evolutionary shift from traditional beat-to-beat morphological classification to complex rhythm pattern recognition. While earlier methodologies primarily focused on isolated 12-lead waveform shapes, recent advancements—particularly those employing Transformer architectures—have increasingly addressed integrated rhythm analysis. Nevertheless, cross-study comparisons remain highly challenging due to significant variations in train/test data splits, selected disease categories, and sample sizes. Therefore, the literature compiled below serves as an objective overview of this transition, highlighting the ongoing effort to balance detailed waveform interpretation with comprehensive rhythm diagnostics.

Continuous home monitoring has become crucial for managing high-risk arrhythmias that require specific medical interventions, such as SVT and VT. Given the unpredictable nature of these cardiac conditions, prompt screening and capture through home-care systems are clinically imperative [1,2]. To address these clinical needs, recent advancements in the past year have highlighted the potential of deep learning in ECG classification. However, due to the inherent complexity of ECG signals and their waveform variations over extended periods, relying solely on traditional CNNs or RNNs remains highly challenging. This limitation necessitates the introduction of Transformer-based architectures, whose self-attention mechanisms overcome the fundamental constraints of CNNs and RNNs by effectively achieving global modeling of long-range dependencies across lengthy ECG records [6,7].

Recent literature reflects this methodological shift. For example, Apostol and Nutu [27] utilized a large-scale 12-lead ECG database to implement an image-based Transformer model, achieving overall F1-scores ranging from 0.79 to 0.84. Similarly, Showrav et al. [3] utilized a large-scale 12-lead database to develop a single-lead classification framework by transforming signals into images and incorporating R-R interval features. This approach effectively distinguished between ECG patterns with overlapping frequency and morphological characteristics, reaching an accuracy over 0.96.

Beyond basic sequential analysis, recent breakthroughs have demonstrated the potential of multi-modal feature fusion within Transformer frameworks. For instance, Yisimitila et al. [8] introduced a Momentum Distillation Transformer (MDOT) that integrates 2D oscillographic representations with clinical features. By leveraging self-attention to capture multi-dimensional dependencies, such architectures achieve superior diagnostic accuracy (up to 99.53%) in complex arrhythmia classification, suggesting that the integration of diverse signal representations is key to robust cardiac modeling.

The practical deployment of AI in home-care settings also hinges on its ability to handle non-standard data and provide ‘clinically meaningful’ interpretability. Gliner et al. [5] recently validated an AI framework capable of analyzing digitized or mobile-captured ECG photos, demonstrating a high correlation between AI-driven insights and the diagnostic logic of expert electrophysiologists. This transparency is particularly crucial for non-specialists managing life-threatening conditions like SVT and VT, as it bridges the trust gap between automated screening and clinical intervention.

Furthermore, current trends involve extracting single-lead data from comprehensive 12-lead databases for specialized analysis. ECG researchers have increasingly focused on single-lead ECGs for both normal rhythm and myocardial infarction (MI) classification. For instance, Ezz [4] demonstrated that MobileNetV2 and VGG16 models can predict MI using single-lead data (specifically lead V4), with F1-scores as high as 0.98. This study successfully validated that single-lead ECG analysis can be effectively implemented in edge-based devices. Ultimately, whether maintaining the data as a 1D time-series or converting it into 2D image-based representations (such as spectrograms), these diverse signal processing strategies have consistently demonstrated their efficacy in clinical diagnostic tasks.

### 1.2. The Objective and Core Innovations of This Study

The objective of this study is to leverage publicly available large-scale data to develop a Transformer-based model that accurately classifies five arrhythmia types: normal sinus rhythm (NSR), ST, sinus bradycardia (SB), SVT, and VT. Future home-based ECG monitoring will enable patients to use simple devices to automatically distinguish between life-threatening and benign issues. In routine clinical practice, a standard diagnostic procedure involves medical personnel placing two electrode patches on a patient’s chest to record a brief 10 s single-lead ECG strip for rapid rhythm assessment.

To address the limitations of earlier studies with insufficient subject numbers, we utilized the PhysioNet/Computing in Cardiology Challenge 2020 (PhysioNet 2020) [29,30] database. Recognizing the low incidence of VT in short-duration recordings, we supplemented our data with the MIT-BIH Malignant Ventricular Ectopy Database (VFDB) [29,31] to ensure adequate representation of sustained VT. By focusing on rhythm-level analysis via 10 s recordings—rather than individual heartbeats—this study replicates this clinical workflow for rapid triage, providing a practical solution for both clinical and home-care applications.

While previous ECG research has predominantly relied on public datasets for beat-to-beat morphological classification or 12-lead waveform recognition, this study targets the future application of home-based ECG monitoring, where single-lead devices are highly prevalent. In this context, the primary objective is to leverage artificial intelligence to minimize computational complexity while ensuring the reliable extraction and recognition of essential ECG features. Unlike traditional approaches that concentrate solely on waveform morphology, this study emphasizes the concurrent identification of both morphology and rhythm. By extracting features through a 1 s sliding window, our model can simultaneously determine both the shape and rhythm of the ECG signal, eliminating the reliance on auxiliary features such as the R-R interval.

The selection of specific cardiac conditions in this study is highly clinically driven. ST and SB represent critical metrics for home-based remote cardiac surveillance, as both rhythms are closely associated with early autonomic nervous system dysfunction. Early detection of these conditions through continuous monitoring can significantly improve clinical outcomes by identifying potential autonomic imbalances before they escalate into more severe cardiovascular events. Conversely, SVT and VT constitute severe events that demand rapid clinical intervention, making their timely detection critical. By utilizing exclusively single-lead signals, this study aims to overcome the diagnostic challenges posed by overlapping frequency ranges and clinically similar ECG features, ultimately contributing to a feasible and effective AI-assisted home telemonitoring system.

To achieve these clinical objectives, the core contributions and innovations of this study are summarized as follows:

1. We developed a lightweight architecture featuring a highly efficient Transformer model comprising approximately 0.7 M parameters (with a reduced model dimension d_model = 256). This architecture automates single-lead ECG analysis, bypassing the need for manual feature extraction processes, such as R-R interval calculation.
2. The model achieves precise frequency distinction by accurately classifying cardiac rhythms that share similar P-QRS-T waveforms but operate at distinctly different heart rates, including normal sinus rhythm, ST, and SB.
3. For advanced pathology identification, the framework effectively differentiates highly confusable conditions that fall within similar heart-rate ranges but possess fundamentally different pathological characteristics, most notably distinguishing between ST, SVT, and VT.
4. To ensure the system is ready for wearable integration, the model was trained extensively on Lead I time-series data. Its low computational cost renders it exceptionally well-suited for real-world deployment in single-lead ECG patches and smartwatches.

## 2. Materials and Methods

The methodology of this study was implemented in two phases. In the first phase, we curated a large-scale ECG dataset by integrating multiple open-source repositories and approached a patient-wise (subject-oriented) splitting to mitigate potential bias from intra-patient correlations and ensure clinical generalizability. This phase involved standardized data preprocessing, including signal resampling, data normalization, and the extraction of lead I ECG (or compatible lead II) signals to align with home-care monitoring scenarios. A 1 s sliding window with a 5-point stride as the input of a DL model. In the second phase, we adopted a lightweight, rhythm-centric Transformer-based architecture (comprising approximately 0.7 million parameters) integrated with Time2Vec embedding. This model was specifically optimized to differentiate five clinically significant heart rhythms, NSR, ST, SB, SVT (incorporating AT), and VT, emphasizing the identification of life-threatening conditions suitable for resource-constrained edge devices.

### 2.1. Dataset Description

To develop and evaluate the proposed model, ECG data were integrated from two prominent open-source repositories: PhysioNet 2020 [29,30] and VFDB [29,31].

**Figure 1:**
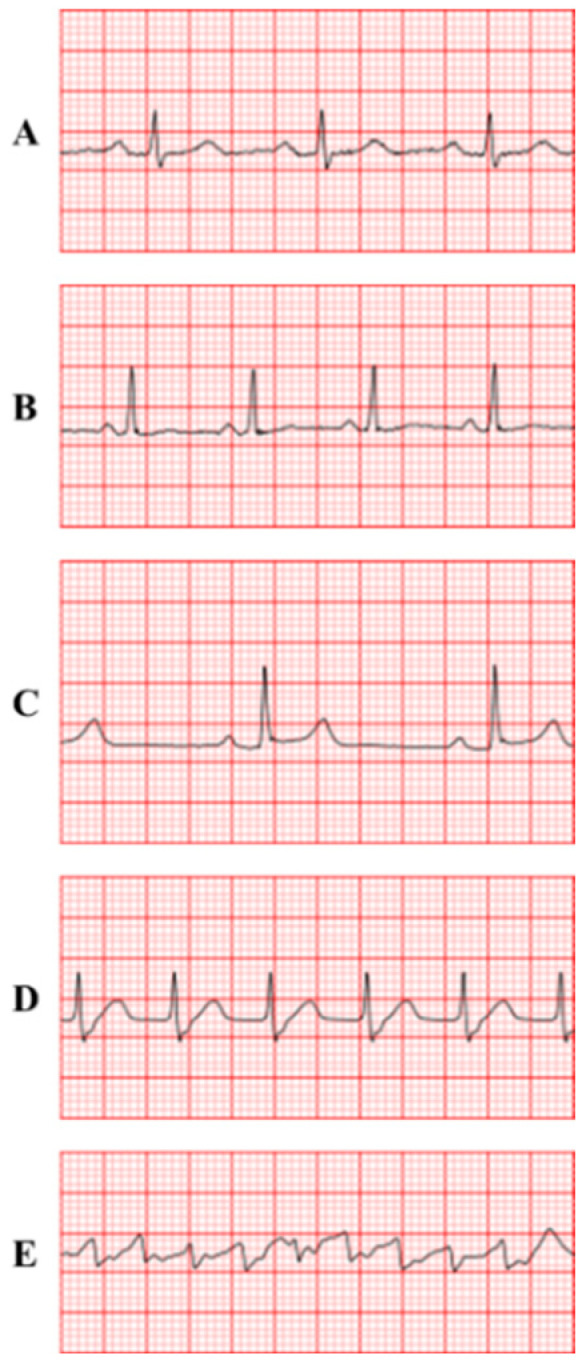
Representative 10-second ECG segments for the five target heart rhythm categories: (**A**) Normal Sinus Rhythm (NSR); (**B**) Sinus Tachycardia (ST); (**C**) Sinus Bradycardia (SB); (**D**) Supraventricular Tachycardia (SVT); and (**E**) Ventricular Tachycardia (VT).

Each sample in this study was labeled with one of the five heart rhythm categories: NSR, ST, SB, SVT, and VT. The model-predicted ECGs of NSR, ST, SB, SVT, and VT are illustrated in Figure 1.

Based on Figure 1, five ECGs (A–E) demonstrate characteristics of sinus rhythms (NSR, ST, SB) and pathological tachycardias (SVT, VT). Sinus rhythms (A, B, C) originate from the sinoatrial node and differ in heart rate. SVT (D) originates from the atria with an often-obscured P wave. VT (E) originates from the ventricles, showing a distorted QRS complex.

#### 2.1.1. Database Specifications

The PhysioNet 2020 dataset provides a diverse collection of 66,361 12-lead ECG recordings sampled at frequencies varying from 257 Hz to 1 kHz with amplitude values represented as integers. Each 10 s ECG recording in this database was obtained from a unique individual, with only a few cases involving multiple recordings from the same subject. Due to the multi-source nature of the database, each 10 s recording was treated as being obtained from a distinct individual where explicit subject identifiers were unavailable. For this study, we extracted the Lead I signals to align with home-care monitoring scenarios.

The VFDB contains 22 half-hour continuous recordings of two-lead ECGs—specifically Modified Lead II and Lead V1—primarily focused on malignant ventricular arrhythmias, sampled at 250 Hz with a 12-bit resolution. To ensure consistent feature representation across different data sources, the Lead II channel from the VFDB was specifically selected to supplement the Lead I recordings from PhysioNet 2020. From an electrophysiological perspective, unlike precordial leads such as V1, both Lead I and Lead II are limb leads that typically record prominent R-wave morphology when the cardiac axis is within the normal range. Given that our rhythm-level analysis prioritizes temporal dependencies (R-R intervals) and macro-morphological changes—such as the distinctive QRS widening observed in VT—the integration of these two limb leads facilitates the development of a more robust, lead-agnostic feature extraction capability. This approach is particularly essential for real-world single-lead home-care monitoring devices, where electrode placement may vary. All signals were resampled to 250 Hz during preprocessing to ensure architectural consistency.

#### 2.1.2. ECG Selection and Labeling

This study primarily focuses on the preliminary exploration of home-based ECG monitoring. At present, the most commonly used devices for home ECG detection are single-lead patch-type monitors (Lead I placement). Compared with 12-lead systems, single-lead ECG patches are more widely adopted in home-care due to their ease of use and high patient compliance, making them the most practical choice for real-world clinical deployment despite providing a more limited range of electrical information. Unlike traditional approaches that concentrate solely on waveform shapes, our study integrates both morphology and rhythm identification to maximize the diagnostic value of single-lead data. However, a notable limitation remains: Lead I is inherently less sensitive to ST-segment elevation (STE). Consequently, while our dual-approach model is robust for rhythm analysis, it may not be suitable for the reliable detection of myocardial infarction (MI) in home-care settings.

**Figure 2:**
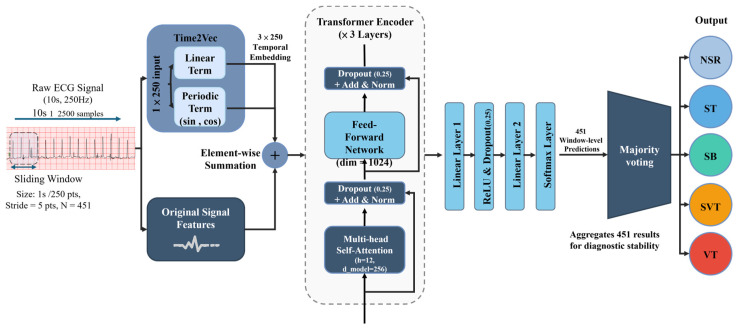
Architectural diagram of the proposed Transformer model. Different colors represent distinct functional stages: pink for raw signal input, dark blue for feature embedding and the Transformer encoder, light blue for linear classification layers, and various colors for the final output classes. Arrows indicate the direction of data flow; the ‘+’ symbol denotes element-wise summation; and the dashed line indicates the scope of the 3-layer Transformer encoder block. The ‘×’ indicates that the encoder layer is repeated three times. Abbreviations: NSR, Normal Sinus Rhythm; ST, Sinus Tachycardia; SB, Sinus Bradycardia; SVT, Supraventricular Tachycardia; VT, Ventricular Tachycardia.

A total of 4297 10 s lead I ECG recordings from the PhysioNet 2020 database, including NSR, ST, SVT, and SB, were utilized for model development. The 10 s VT samples were obtained based on the annotations in VFDB. As illustrated in Figure 2, each 10 s recording was processed into 1 s sliding windows with a 5-point stride. This windowing strategy resulted in a sequence of sub-segments that capture fine-grained morphological changes while maintaining the temporal context of the 10 s rhythm.

The criteria for inclusion required that each 10 s segment contains a consistent rhythm corresponding to one of the five target classes: NSR, SB, ST, SVT, or VT. While recordings with ambiguous clinical labels or overwhelming corruption were excluded to ensure the reliability of the ground truth, we did not deliberately select only “clean” signals. To better reflect real-world clinical scenarios, samples containing typical high- or low-frequency noise, motion artifacts, or baseline wander were retained, provided that human experts could still visually identify the underlying cardiac conditions. All samples were chosen randomly to ensure the model learns robust pattern recognition against typical ECG noise.

To address the severe class imbalance inherent in the massive original PhysioNet database, we performed random sampling to equalize the number of samples across classes. A training set of 1500 records was compiled (comprising exactly 300 recordings per targeted class), and a validation set of 500 records was created. A separate 2297-record testing set was used to assess the model’s generalizability. This deliberate dataset reduction and balancing strategy prevents the model from being heavily biased toward majority classes, thereby fully preserving its learning potential and sensitivity to minority yet clinically critical classes. Table 2 provides a detailed summary of the data distribution, with the number of patients for PhysioNet 2020-derived classes estimated based on the total number of recordings to mitigate subject-level bias.

#### 2.1.3. Patient-Wise Data Partitioning

To ensure the robustness of the inter-patient evaluation, the dataset partitioning was approached through a patient-wise (subject-oriented) strategy to divide the records into training, validation, and testing sets. Specifically, each ECG recording derived from a single patient was assigned to only one of these three subsets, with a necessary exception for the VT class due to limited subject availability. This approach prevents “data leakage” across the primary dataset, where the model might otherwise memorize a specific patient’s wave morphology. The distribution of patients and ECG recordings across classes is detailed in Table 1.

### 2.2. Model Architecture

We developed a Transformer-based model optimized for the discrimination of five clinically significant heart rhythms: NSR, ST, SB, SVT (including AT), and VT. To ensure suitability for resource-constrained home-care devices, the proposed Transformer architecture was specifically optimized for computational efficiency. As illustrated in Figure 2, the model employs a streamlined 3-layer Transformer encoder with a significantly reduced model dimension (d_model = 256). This architectural refinement minimizes the memory footprint while maintaining the core representational power required for complex ECG rhythm analysis.

The Transformer architecture was selected for its capability to capture long-range temporal dependencies within ECG sequences via self-attention mechanisms. To determine the optimal configuration, a grid search-based hyperparameter optimization was performed, resulting in the final parameters illustrated in Figure 2. The architecture comprises four primary components: Temporal Feature Embedding via Time2Vec, the Transformer encoder, the arrhythmia classification output layer, and a decision mechanism based on majority voting.

The proposed architecture accepts standardized temporal sequences as input. The specific data preprocessing and windowing strategies used to generate these inputs are detailed in Section 2.3.

#### 2.2.1. Temporal Feature Embedding via Time2Vec

The positional encoding part of the original transformer model was replaced with Time2Vec. The learnable temporal embedding layer employs the Time2Vec module rather than non-learnable positional encoding. This choice is justified by recent architectural benchmarks in time-series forecasting [32,33]. While standard Transformers use fixed sine/cosine functions for natural language processing and image processing, Time2Vec introduces learnable parameters that allow the model to adapt to the specific periodicities and trends inherent in the quasi-periodic time-series dataset, such as ECG waveforms. Previous studies in similar domains have demonstrated that this adaptive embedding significantly improves the model’s ability to handle non-stationary data and long-range temporal dependencies, providing a more robust foundation for the self-attention mechanism without the need for manual feature engineering [33,34]. Furthermore, because Time2Vec has been clearly established as a superior alternative to fixed positional encoding for ECG-like signals in recent literature, it was selected as a core component of our architecture to ensure optimal feature extraction. As illustrated in the block-diagram within Figure 2, the input to this block is a 10 s ECG recording, which is segmented into 1 s sliding windows with a 5-point stride. Time2Vec transforms time-series data into vector representations [35]. For our particular input signal, the Time2Vec block generates an output dimension of 3 ×250, which effectively captures both the periodicity and non-periodicity of ECG signals. The mathematical expression for Time2Vec is given by Equation (1) [36]: $t2v\left(\tau \right)$ is a (***k*** + 1)-dimensional vector and $t2v\left(\tau \right)\left[i\right]$ is the *i*-th element of $t2v\left(\tau \right)$. F is a periodic activation function, $\omega_{i}$ and $\varphi_{i}$ are both learnable parameters, $\omega_{i}$ is used to capture periodic patterns, $\varphi_{i}$ is used to capture non-periodic patterns, and $\omega_{i}\tau +\varphi_{i}$ represents non-periodic time features. We employed the sine function as the periodic activation function because it is most effective in capturing periodic behavior and requires no additional feature engineering.

(1) $$t2v\left(\tau \right)\left[i\right]=\left\{\begin{array}{c} \omega_{i}\tau +\varphi_{i},\;\;\;\mathrm{if}\;i=0.\\ F\left(\omega_{i}\tau +\varphi_{i}\right),\;\;\;\mathrm{if}\;1\leq i\leq k \end{array}\right.$$

#### 2.2.2. Transformer Encoder

The input of the “Transformer encoder” block is the element-wise summation of the vectorized ECG sub-segments and the temporal embeddings generated by the Time2Vec block. Consistent with our focus on computational efficiency, we built our DL model using three identical encoder layers, each with two sublayers. The first sublayer is a multi-head self-attention mechanism with 12 attention heads, designed to capture long-range dependencies in the input time series. The second is a fully connected feed-forward neural network with a dimension of 1024 that processes the attention output. To prevent overfitting, we included a dropout layer (rate: 0.25) and applied L2 regularization (0.001). Both sublayers use residual connections and layer normalization. The specific parameters used in this structure, such as a reduced d_model of 256, were selected based on hyperparameter optimization to ensure model accuracy while achieving an exceptionally lightweight parameter count (approximately 0.7 M).

Self-attention mechanisms are designed to effectively capture the interdependencies among different positions within an input sequence of time vectors, such as those derived from ECG signals. These interdependencies are quantified through attention scores, which reflect the relevance of each position relative to others in the sequence. In this study, the scaled dot-product attention method was employed to integrate the query (*Q*), key (*K*), and value (*V*) vectors via matrix operations, as detailed in Equation (2) [36]. Specifically, *Q* encapsulates the information to be retrieved from the sequence, *K* encodes the features of each sequence element, *V* represents the actual content associated with each element and $\sqrt{d_{k}}$ scales the dot product to prevent large values, enabling the Transformer to focus on critical sequence parts. The self-attention mechanism finds the most relevant value vectors by comparing the query vector with the key vector and then performs a weighted sum of these value vectors to obtain the final output. This enables the transformer model to automatically focus on the most important parts of the sequence, thereby improving its performance.

(2) $$Attention\left(Q,K,V\right)=softmax\left(\frac{QK^{T}}{\sqrt{d_{k}}}\right)V$$

When multiple query, key, and value vectors are used to capture different information within an input sequence, a multi-head self-attention mechanism is formed. The self-attention mechanism was applied h times, and the outputs were concatenated. Therefore, each head is mapped to a different weight. Because each head focuses on different types of information, the model can obtain a more comprehensive understanding. The mathematical expression for this is shown in Equation (3) [36].

(3) $$\begin{array}{c} MultiHead\left(Q,K,V\right)=Concat\left({head}_{1},{head}_{2},\ldots {head}_{h}\right)W^{0}\\ where\;{head}_{i}=Attention\left(QW_{i}^{Q},KW_{i}^{K},VW_{i}^{V}\right) \end{array}$$

The fully connected feed-forward neural network (FFN) layer was independently applied to the input vector, followed by processing through two one-dimensional convolutional layers activated by a rectified linear unit (ReLU). This layer transforms the output of the preceding multi-head self-attention mechanism into a higher-dimensional feature space, enabling the extraction of enriched feature information critical for ECG signal classification. The mathematical expression governing this transformation is presented in Equation (4) [36], where *x* denotes the input vector to the neural network, *W* represents the trainable weight matrix, *b* signifies the bias vector, and $FFN\left(x\right)\;$ is the output of the layer after applying the ReLU activation function.

(4) $$FFN\left(x\right)=\max\left(0,xW_{1}+b_{1}\right)W_{2}+b_{2}$$

To address the issue of DL networks becoming unable to learn effectively beyond a certain depth, the residual connections are applied to add the input and output of two sublayers as illustrated in Figure 2, preventing information loss and enabling the addition of deeper networks to improve model accuracy. Layer normalization processes the different dimensions of the same feature vector. Specifically, it calculates the mean and standard deviation of the input vector for that feature and normalizes the output vector of that feature accordingly. This method is suitable for recurrent networks and for sequential data. The mathematical expression for this is shown in Equation (5) [36].

(5) $$LayerNorm\left(x+Sublayer\left(x\right)\right)$$

#### 2.2.3. Arrhythmia Classification Output Layer

The linear layer is a fully connected layer that maps the final output of the Transformer encoder to a logit vector *z*. As illustrated in Figure 2, we employed two consecutive linear layers separated by a ReLU activation function and a dropout layer. This non-linear configuration prevents the two layers from being mathematically equivalent to a single linear layer, thereby enhancing the model’s ability to learn complex feature mappings. The softmax function is a normalization exponential function layer which transforms the logits *z* into a probability distribution as shown in Equation (6): here, $p_{i}$ is the probability value for the *i*-th class, $z_{i}$ is the logit value for the *i*-th class, the probability value for each class lies within the range [0, 1], and the sum of probabilities for k classes equals one.

(6) $$p_{i}=\frac{e^{z_{i}}}{\sum_{j=1}^{k}e^{z_{j}}}\;for\;i=1,\;2,\;\ldots ,\;k$$

#### 2.2.4. Decision Mechanism: Majority Voting

Because we used multiple sliding windows to analyze 10 s ECG signals, the same ECG can generate different classification labels. We utilize a voting mechanism to obtain the most frequent classification label as the final classification output for the ECG. Crucially, this mechanism provides a necessary safeguard against the interference of localized ECG noise within the 10 s window. Even if a few noisy segments are misclassified due to high- or low-frequency artifacts, the overall 10 s prediction can still remain accurate, reflecting the robustness required for real-world clinical monitoring.

This voting mechanism is known as majority voting and its mathematical expression is given by Equation (7). In Equation (7), $\hat{y}$ denotes the final predicted rhythm class for the 10 s ECG recording. *m* represents the total number of sliding 1 s windows derived from a 10 s ECG recording, and *j* = 1, …, *m* is the index of each window. The term $\sum_{j=1}^{m}1\left(C_{j\left(x\right)}=\;c\right)$ counts how many times a specific class *c* (where $c\;\in \left\{NSR,\;ST,SB,\;SVT,\;VT\right\}$) appears across all *m* windows. The argmax function then selects the class c with the highest frequency as the final output.

(7) $$\hat{y}=argmax_{c\;\in \left\{1,\;\ldots ,\;K\right\}}\sum_{j=1}^{m}1\left(C_{j\left(x\right)}=c\right)$$

The proposed models were implemented using Python (version 3.9.2, Python Software Foundation, Wilmington, DE, USA) on an Ubuntu 20.04.3 LTS operating system (Canonical Ltd., London, UK). Hardware included an Intel Core i5-7500 CPU (Intel Corp., Santa Clara, CA, USA) and an NVIDIA GeForce GTX 1050 Ti GPU (NVIDIA Corp., Santa Clara, CA, USA). The deep learning environment was supported by CUDA (version 11.3.1), cuDNN (version 8.2.1), and TensorFlow (version 2.6.5, Google LLC, Mountain View, CA, USA).

### 2.3. Data Preprocessing

Following the architectural presentation of the model, this section clarifies the localized mathematical operations used to prepare the input layer.

#### 2.3.1. Signal Normalization

Each 10 s signal was normalized using Z-score normalization to mitigate amplitude variations across different recording devices and lead configurations. The normalization is expressed as:

(8) $$\tau_{i}=\frac{X_{i}-\bar{X}i}{\sigma_{i}}$$

In this formulation, $X_{i}$ represents the entire sequence of the *i*-th 10 s ECG segment (treated as a vector). Crucially, the mean ($\bar{X}i$) and standard deviation ($\sigma_{i}$) were calculated based on the full 10 s interval rather than shorter windows. This approach preserves the relative morphological features, such as the R-wave height relative to the T-wave, within each specific rhythmic context.

#### 2.3.2. Input Layer Specifications

The resulting input to the network is a single-channel 10 s duration signal unified at a sampling rate of 250 Hz, totaling 2500 samples. As illustrated in Figure 2, this 10 s segment is further processed into 1 s sliding windows with a 5-point stride.

The selection of the 1 s window length (equivalent to 250 data points) is primarily based on the physiological characteristics of the ECG signal. For example, when identifying conditions such as sinus bradycardia (<60 beats/min) or tachycardia (>100 beats/min), a 1 s window is sufficient to distinguish the relevant cardiac intervals, making it an optimal unit for analysis. Furthermore, the 5-point stride serves as our primary data augmentation strategy, determined by the memory capacity of computers typically used in medical facilities and our target design goal of maintaining a lightweight architecture with approximately 0.7 million parameters. This windowing strategy transforms the 10 s rhythm into a sequence of 451 sub-segments, each containing 250 data points. As this study utilized only 300 original recordings per class to maintain strict class balance and prevent majority-class bias, the multi-window mechanism becomes a critical prerequisite for model convergence. Consequently, for a single disease category containing 300 original ECG records, this approach expands the dataset to 135,300 window samples.

This substantially augmented data volume effectively mitigates the risk of “data starvation” and overfitting, inherently demonstrating the absolute necessity of this multi-window mechanism for training our Transformer model. Therefore, a traditional ablation study removing this mechanism is considered impractical; without this data augmentation, the model would fail to train effectively due to the limited initial sample size, rather than providing insights into architectural dynamics. This representation allows the model’s input layer to capture detailed morphology within each 1 s window and the broader temporal relationships across the 10 s rhythm.

### 2.4. Model Performance Evaluation

In medical diagnostics, true negative (TN), true positive (TP), false negative (FN), and false positive (FP) describe test outcomes. A TP is a correct positive result, and a TN is a correct negative result. An FP occurs when a test is positive for an individual without the condition, while an FN occurs when a test is negative for someone with the condition. We assessed our model’s performance using Accuracy (Acc), Sensitivity (Se), Specificity (Sp), Precision (Pre), F1-score (F1), and Area Under the Curve (AUC).

Accuracy refers to the proportion of correct predictions among all samples. It is the ratio of correctly identified samples to the total number of samples. The calculation formula is shown in Equation (9):

(9) $$Acc=\frac{TP+TN}{TP+TN+FP+FN}\times 100\%$$

Sensitivity is also known as the proportion of actual positive samples that are correctly identified as positive. It measures the model’s ability to find all positive samples. The calculation formula is shown in Equation (10):

(10) $$Se=Recall=\frac{TP}{TP+FN}\times 100\%$$

Specificity refers to the proportion of actual negative samples that are correctly identified. It measures the model’s ability to correctly identify all negative samples. The calculation formula is shown in Equation (11):

(11) $$Sp=\frac{TN}{TN+FP}\times 100\%$$

Precision refers to the proportion of predicted positive samples that were actually positive. The calculation formula is shown in Equation (12):

(12) $$Pre=\frac{TP}{TP+FP}\times 100\%$$

The F1-score is a balanced measure of overall model performance, considering both sensitivity and precision. The calculation formula is shown in Equation (13):

(13) $$F1=\frac{2\times Sen\times Pre}{Sen+Pre}\times 100\%$$

ROC curve plots Sensitivity (True Positive Rate) against 1-Specificity (False Positive Rate). The closer the curve is to the top-left corner, the better the model’s predictive power. AUC, which is the area under this curve, ranges from 0 to 1. An AUC > 0.5 indicates performance better than random guessing. An AUC > 0.8 is considered good, and >0.9 is excellent. An AUC of 1.0 indicates perfect predictive power.

## 3. Results

**Figure 3:**
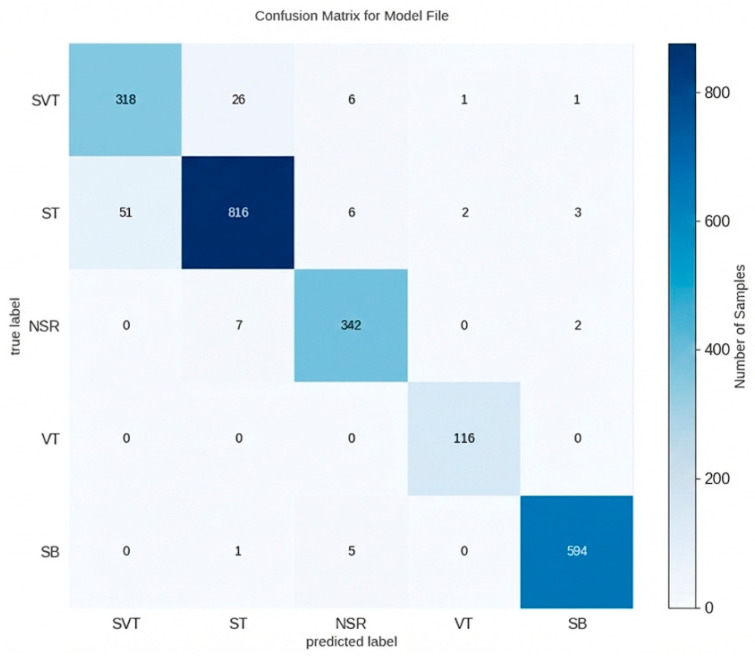
Confusion matrix results on the test dataset.

The confusion matrix in Figure 3 shows the model’s accurate performance across all five categories. The majority of samples are correctly classified along the diagonal. The intensity of the color tones corresponds to the absolute number of samples in each category, where the variations in depth along the diagonal reflect the original distribution and relative scales of the source databases, such as PhysioNet 2020 and VFDB. This visualization clearly distinguishes correct predictions from incorrect ones, with the concentration of samples on the diagonal demonstrating high classification accuracy regardless of the specific sample volume. The model performed particularly well in identifying NSR (342 correct recordings), SB (594 correct recordings), and VT (116 correct recordings, with no major misclassifications). The main confusion was observed between SVT and ST (SVT misclassified as ST 26 times, and ST misclassified as SVT 51 times), with minor confusion between NSR and SB (NSR misclassified as SB 2 times, and SB misclassified as NSR 5 times). Overall, the distribution of numerical values and the corresponding gray levels demonstrate that the model reliably distinguishes the five rhythm categories with minimal cross-class confusion.

As summarized in Table 3, the proposed model achieved an overall accuracy of 95.2%. It demonstrated high accuracy across all classifications, with the highest for VT (99.9%), followed by SB (99.5%), NSR (98.9%), SVT (96.3%), and ST (95.8%). The model also showed high sensitivity for clinically fatal VT (100%) and achieved over 90.3% sensitivity for SVT. With an F1-score of approximately 88.2% for SVT, the model showed robust performance, with other classifications also performing well.

To further evaluate the model’s discriminative ability, the Area Under the Curve (AUC) for each class was calculated, as presented in Table 3. The AUC values for all analyzed arrhythmias exceeded 0.98, indicating excellent classification performance. Although the AUC for SVT was slightly lower at 0.978, the model maintains high sensitivity while effectively minimizing false-positive rates. These consistent results across multiple metrics confirm the feasibility of the proposed Transformer-based architecture for reliable arrhythmia detection.

## 4. Discussion

### 4.1. The Importance of Rhythm-Level Analysis in Clinical Diagnosis

A key aspect of this research is the transition from individual “single-beat morphology” to comprehensive “rhythm-level analysis” for arrhythmia detection. While much of the foundational ECG AI literature focuses on classifying individual QRS complexes—often following the AAMI standard—clinical diagnosis of life-threatening conditions like SVT and VT inherently necessitates a broader temporal context.

Fatal arrhythmias are clinically defined by their progression over time rather than isolated wave distortions. By employing 10 s ECG segments, our model identifies rhythmic patterns that may be less apparent in beat-by-beat analysis. This segment-based approach is particularly effective in distinguishing arrhythmias with overlapping morphologies but distinct temporal signatures, such as ST versus SVT. Our findings demonstrate that the Transformer’s self-attention mechanism, augmented by Time2Vec embedding [35], identifies these complex patterns without manual interval measurements, offering a more robust framework for real-time monitoring.

### 4.2. Methodological Advantages over Traditional Analysis

The effective discrimination between ST, SB, and NSR in this study highlights a significant methodological advantage over traditional machine-learning approaches. Historically, the accurate classification of these rhythms—which share similar P–QRS–T morphological features but differ in underlying frequency—required precise computation of R–R intervals and meticulous verification of complete P-QRS-T complexes. While such assessments are straightforward for clinicians, they present substantial computational challenges for automated systems in manual feature engineering. Unlike these traditional methods, our study adopts a “Dual Approach” that integrates both morphology and rhythm identification. For instance, the model successfully differentiates conditions that share similar key ECG components but exhibit distinct rhythm patterns, such as NSR, ST, and SB. Similarly, our approach addresses arrhythmias that may fall within overlapping heart-rate ranges yet present with different morphological characteristics, such as SVT, ST, and VT.

In this study, we adopted a simplified sliding-window approach that bypasses the explicit calculation of R–R intervals or the structural confirmation of individual waveforms. Despite this streamlined strategy, the Transformer-based model achieved exceptional accuracy across these categories. This demonstrates that the model can autonomously learn representative temporal dependencies, offering a more efficient and robust alternative to conventional beat-to-beat analysis methods for real-time home-care monitoring.

### 4.3. Data Integration Strategy and Representativeness

The scarcity of VT samples in open-source databases remains a challenge for training robust DL models. By supplementing our dataset with the MIT-BIH Malignant Ventricular Ectopy Database (VFDB), we aimed to ensure high sensitivity for these rare but fatal events. We acknowledge that the concentrated nature of the VFDB (comprising 16 subjects) may influence the high detection metrics observed for VT; however, this targeted strategy is a critical step in developing early-warning systems for high-risk patients. Furthermore, the integration of SVT and Atrial Tachycardia (AT) categories was a deliberate clinical choice based on their aligned management protocols in emergency settings, allowing for more efficient feature extraction within the model.

### 4.4. Pathological Mechanisms and Interpretability Limitations

While the model achieved high overall efficacy, we observed a certain level of classification confusion between ST and SVT. Physiologically, this can be attributed to overlapping heart rate ranges and the “P-on-T” phenomenon, where P-waves become obscured by preceding T-waves, making morphological distinction challenging. While feature visualization has progressed, it remains insufficient for translating AI predictions into clinical reasoning [5,8,28]. Model-agnostic tools like SHAP and LIME frequently fail to provide the morphological precision necessary for ECG interpretation, often attributing importance to broad waveforms rather than specific diagnostic features. Furthermore, the architectural nature of image-based Grad-CAM—specifically the resolution loss inherent in downsampling and pooling—results in coarse heatmaps that can overlook subtle, high-frequency signals. Given the dual challenges of morphological and rhythmic variation in our time-series 5-class classification task, relying solely on these visualizations is problematic for mechanistic analysis. Nevertheless, an in-depth investigation of these limitations is a vital precursor to the development of next-generation, interpretable cardiac AI aimed at minimizing such confusion and reducing false alarm rates.

### 4.5. Computational Efficiency and Hardware Feasibility

A critical requirement for home-based ECG monitoring is the ability to operate on hardware platforms with limited resources, such as mobile and wearable devices. To address these demands, our architecture employs a streamlined 3-layer Transformer encoder with a reduced model dimension (d_model = 256), as described in Section 2.2. This optimized configuration achieves an efficient parameter count of approximately 0.7 M. Compared to standard mobile-optimized benchmarks—such as the MobileNetV3 family (1.9 M to 10.5 M parameters) [37]—our model is significantly more lightweight. This substantial reduction in structural complexity ensures that the proposed system meets clinical requirements for real-time operation and low detection latency on target edge hardware. Furthermore, the integration of Time2Vec and self-attention mechanisms provides high diagnostic accuracy without the prohibitive computational overhead typically associated with standard Transformer models, confirming the feasibility of the proposed solution for practical telemonitoring applications.

### 4.6. Limitations and Future Directions

While the proposed model demonstrates high efficacy, certain clinical limitations must be acknowledged. Specifically, Lead I is inherently less sensitive to ST-segment elevation (STE); thus, the model may not be suitable for the reliable detection of myocardial infarction (MI) in home-care settings. Furthermore, external validation on independent clinical datasets remains a priority to further mitigate potential overfitting. Future work will explore multi-lead fusion and enhanced interpretability frameworks [4] to address these challenges.

## 5. Conclusions

In this study, we developed a lightweight model utilizing a single-lead (Lead I) ECG to reliably detect key arrhythmias, including Sinus Tachycardia (ST), Sinus Bradycardia (SB), Supraventricular Tachycardia (SVT), and Ventricular Tachycardia (VT). Our findings demonstrate that a simple single-lead device, powered by our efficient architecture, can serve as a highly practical tool for home-based cardiac monitoring. Clinically, this system facilitates both the early detection of abnormalities linked to autonomic nervous system disorders (e.g., ST and SB) and the rapid identification of severe arrhythmias that require immediate medical intervention (e.g., SVT and VT). By combining computational efficiency with high diagnostic accuracy, the proposed framework provides a feasible solution for real-world deployment in wearable telemonitoring devices.

## Figures and Tables

**Table 1:** Summary of related research on ECG classification and analysis.

Data Source	Input Features	Network Architecture	Classes	Performance	References
MIT-BIH Arrhythmia Database	single QRS beat	PCA-based Transformer Encoder	5 (Normal, APC, VPC, Fusion beat and Others)	Acc of all = 97.10% F1 = 95.00%	Ikram et al., 2025 [7]
MIT-BIH Arrhythmia Database & PhysioNet/Computing in Cardiology (CiC) Challenge 2017	10 s ECG sequences	DeepECG-Net (Hybrid CNN–Transformer)	2 (Normal vs. Abnormal/Anomaly detection)	Acc of all = 98.30% Se = 96.50% Pre = 97.10% F1 = 96.50%	Alghieth 2025 [6]
PTB-XL, PTB Diagnostic, China 12-Lead, Georgia 12-Lead and St. Petersburg INCART	2D grayscale ECG images (converted from 12-lead signals)	2D Pre-trained Vision Transformers (ViT, BEiT, DeiT)	5 (AF, IAVB, SB, NSR, ST)	ViT pretrained: Acc = 84.60% F1 = 0.84 BeiT_pretrained: Acc = 81.89% F1 = 0.79 DeiT pretrained: Acc = 84.60% F1 = 0.81	Apostol and Nutu 2025 [27]
PhysioNet/Computing in Cardiology (CiC) Challenge 2021 (Chapman and Ningbo datasets)	10 s ECG sequences	EXGnet (CNN + Multiresolution block + XAI guidance)	Chapman: 5 (SR, SB, ST, AF + AFib, SVT + AT) Ningbo: 5 (SR, SB, ST, AF + AFib, SA)	Chapman: Acc = 98.76% Se = 97.85% Spe = 99.70% F1 = 97.91% Ningbo: Acc = 96.93% Se = 95.40% Spe = 99.21% F1 = 95.53%	Showrav et al., 2025 [3]
A publicly available ECG Images Dataset (Mendeley Data) comprising 928 clinical ECG recordings from cardiac patients	2D images of segmented single-lead ECG signals (Lead V4 was identified as the optimal diagnostic lead)	A comparative evaluation of deep CNN architectures, including VGG16, MobileNetV2, InceptionV3, DenseNet201, and NASNetLarge	4 (Normal, Abnormal/Arrhythmia, MI and PMI)	VGG16: F1 = 98.11%, Prediction time = 4.2 ms MobileNetV2: F1 = 97.24%, Prediction time = 3.2 ms	Ezz 2025 [4]
MIT-BIH Arrhythmia Database & Chapman-Shaoxing dataset	2D oscillographic representations (converted from 1D ECG signals via the OSC module) and auxiliary clinical features (including heart rate, QRS duration, ST segment changes, and QTc interval).	MDOT (Momentum Distillation Oscillographic Transformer)	MIT-BIH: 8 Chapman: 12	MIT-BIH: Acc = 99.53% F1 = 97.26% Chapman: Acc = 99.03% F1 = 96.38%	Yisimitila et al., 2025 [8]
NYU dataset (clinical ECGs) and a dataset of scanned or photographed ECG images	Digitized 12-lead ECG images, including scanned ECG images and images obtained via mobile camera in clinical settings	ECG-AIO, an ensemble of deep learning models (specifically ResNet-18-based architectures)	8 (AFib, AF, SB, ST, LBBB, LVH, PVC and RBBB)	Specific numerical metrics not reported; achieved high correlation with gold standard interpretations of 3 electrophysiologists.	Gliner et al., 2025 [5]
PhysioNet/Computing in Cardiology (CiC) Challenge 2020	10 s ECG sequences	ST-CNN-5: a deep Convolutional Neural Network (CNN) architecture focused on interpretable AI (XAI) using Grad-CAM	5 (Normal, MI, STTC, CD and HYP)	Acc of all = 89.10% Pre of all = 79.80% Senof all = 69.30% Spe of all = 93.40%	Ojha et al., 2024 [28]

**Table 2:** Distribution of 10 s ECG segments and patients across arrhythmia classes.

Arrhythmia Class	Estimated No. of Patients	No. of 10 s ECG Recordings	Training Dataset	Validation Dataset	Test Dataset
NSR	751	751	300	100	351
ST	1278	1278	300	100	878
SB	1000	1000	300	100	600
SVT (including AT)	952	952	300	100	352
VT	16	516	300	100	116
Total	3997	4297	1500	500	2297

Note: NSR = Normal Sinus Rhythm; ST = Sinus Tachycardia; SB = Sinus Bradycardia; SVT = Supra ventricular Tachycardia; AT = atrial tachycardia; VT = Ventricular Tachycardia.

**Table 3:** Model evaluation results on the test dataset.

Classification Performances of SVT, ST, NSR, VT and SB.						
	Accuracy	Sensitivity	Specificity	Precision	F1-Score	AUC
SVT	96.3%	90.3%	97.4%	86.2%	88.2%	0.978
ST	95.8%	92.9%	97.6%	96.0%	94.4%	0.987
NSR	98.9%	97.4%	99.1%	95.3%	96.3%	0.997
VT	99.9%	100.0%	99.9%	97.5%	98.7%	1.000
SB	99.5%	99.0%	99.6%	99.0%	99.0%	0.999
Total	95.2%	-	-	-	-	-

Note: NSR = Normal Sinus Rhythm; ST = Sinus Tachycardia; SB = Sinus Bradycardia; SVT = Supraventricular Tachycardia; VT = Ventricular Tachycardia.

## Data Availability

Publicly available datasets were analyzed in this study. The data presented in this study are openly available in the PhysioNet repository: the PhysioNet/Computing in Cardiology Challenge 2020 database at [https://physionet.org/content/challenge-2020/ (accessed on 4 March 2026)] and the Malignant Ventricular Ectopy Database (VFDB) at [https://physionet.org/content/vfdb/ (accessed on 4 March 2026)]. All processed results and model configurations used for analysis are available from the corresponding author upon reasonable request.

## Abbreviations

The following abbreviations are used in this manuscript:

AAMI	Association for the Advancement of Medical Instrumentation
AFib	Atrial Fibrillation
AF	Atrial Flutter
AI	Artificial Intelligence
AT	Atrial Tachycardia
AUC	Area Under the Curve
AVB	Atrioventricular Block
APC	Atrial Premature Contraction
BEiTs	BERT pre-training of image transformers
CD	Conduction Disturbance
CLSM	Central-towards LSTM Supportive Model
CNN	Convolutional Neural Network
CPSC	China Physiological Signal Challenge
DeiTs	Data-efficient image Transformers
DL	Deep Learning
DNN	Deep Neural Network
ECG	Electrocardiogram
FFNN	Feed-Forward Neural Network
FN	False Negative
FP	False Positive
Grad-CAM	Gradient-weighted Class Activation Mapping
GRU	Gated Recurrent Unit
HYP	Hypertrophy
IAVB	First-Degree Atrioventricular Block
LBBB	Left Bundle Branch Block
LSTM	Long Short-Term Memory
LVH	Left Ventricular Hypertrophy
MI	Myocardial Infarction
NSR	Normal Sinus Rhythm
PAC	Premature Atrial Contraction
PMI	Previous Myocardial Infarction
PVC	Premature Ventricular Contraction
QRS	QRS complex
QT	QT interval
RBBB	Right Bundle Branch Block
ROC	Receiver Operating Characteristic
SA	Sinus Arrhythmia
SB	Sinus Bradycardia
SR	Sinus Rhythm
ST	Sinus Tachycardia
STD	ST-segment Depression
STE	ST-segment Elevation
STTCs	ST-segment and T Wave Changes
SVT	Supraventricular Tachycardia
TN	True Negative
TP	True Positive
VFDB	Malignant Ventricular Ectopy Database
ViT	Vision Transformers
VPC	Ventricular Premature Contraction
VT	Ventricular Tachycardia
